# Relationships Between Bronchodilator Reversibility and Mannitol Airway Hyperresponsiveness in Severe Asthma

**DOI:** 10.1002/clt2.70188

**Published:** 2026-07-06

**Authors:** Philipp Suter, Robert Greig, Rory Chan, Brian J. Lipworth

**Affiliations:** ^1^ Scottish Centre for Respiratory Research Ninewells Hospital and Medical School University of Dundee Dundee UK

**Abbreviations**
ACQ: asthma control questionnaire
AHR: airway hyperresponsiveness
AMP: adenosine 5′ monophosphate
ASM: airway smooth muscles
AX: area of reactance
BDR: bronchodilator reversibility
FEF25‐75: forced expiratory flow rate between 25% and 75% of forced vital capacity
FeNO: fractional exhaled nitric oxide
FEV1: forced expiratory volume in 1 s
FVC: forced vital capacity
ICS: inhaled corticosteroids
MART: maintenance and reliever therapy
PD10: provocative dose that causes a 10% decrease in FEV1
R20: resistance at 20 Hz
R5: resistance at 5 Hz
R5–R20: difference between resistance at 5 and 20 Hz
T2: Type 2

To the Editor,

Assessment of asthma includes symptom burden, exacerbation risk and functional evaluation. Functional assessment in asthma may include bronchodilator reversibility (BDR) and airway hyperresponsiveness (AHR). Although both are used to characterise variable airflow limitation, they reflect distinct underlying pathological mechanisms which are interlinked but may not necessarily align within individual patients [1].

BDR reflects the capacity of the airways, predominantly airway smooth muscle (ASM), to dilate in response to short‐acting beta‐agonists. Typically assessed by spirometry as the change in forced expiratory volume in 1 s (FEV_1_). However, alternative methods, such as oscillometry, appear to be more sensitive to BDR than spirometry [2].

AHR can be assessed by either direct or indirect challenge tests. Mannitol acts as an indirect challenge, increasing periciliary liquid osmolarity to induce mast cell degranulation and synthesis of bronchoconstricting mediators, alongside eosinophil recruitment [3]. Indirect challenges appear to relate more closely to eosinophilic airway inflammation and corticosteroid sensitivity than direct challenge testing [4]. AHR is defined as the provocative dose of mannitol required to produce a pre‐specified fall in FEV1, expressed on a log dose‐response scale [5]. A 10% fall (PD10) was used in the two analysed prospective studies, given all patients exhibited severe asthma. This cutoff was considered safer and more sensitive for this vulnerable population.

We undertook a post hoc analysis of a combined cohort of two clinical trials to evaluate the relationship between BDR and AHR [5, 6]. To our knowledge, this is the first study to investigate this relationship specifically in patients with severe, uncontrolled asthma, using indirect bronchial challenge with mannitol.

We conducted a retrospective analysis of patients who demonstrated positive AHR defined as mannitol PD10 FEV1 of < 635 mg, who underwent BDR to salbutamol 400 μg prior to starting biologics. Fractional exhaled nitric (FeNO) measurement (NIOX, Oxford, United Kingdom) followed by oscillometry (Thorasys Tremoflo, Montreal, Canada) and spirometry (Micromedical, Chatham, United Kingdom) were performed in accordance with European Respiratory Society guidelines. BDR was calculated as the difference between post and pre‐bronchodilator measurements and relative change. Correlation analyses were assessed using Pearson's correlation coefficient with nominal two‐tailed *p* values reported as < 0.05, < 0.01, or < 0.001. Statistical analyses were performed using SPSS version 30 (IBM Corp., Armonk, NY, USA).

We included 37 patients (16 females), with mean baseline values (SEM) as follows: age 51.9 years (2.4), body mass index 30.24 kg/m^2^ (0.84), eosinophils 512 cells/mL (43), FeNO 66.1 ppb (8.4), FEV_1_% predicted 81.0% (2.67), FEV_1_/FVC 65.14% (1.30), FEF_25‐75_ 40.16% (2.30), R5‐R20 0.14 kPa/L/s (0.02), AX 2.47 kPa/L (0.37), asthma control questionnaire (ACQ) 2.7 (0.16), and mannitol PD10 130.5 mg (19.53). Mean (SEM) relative % BDR were 9.3% (1.4) FEV1, 24.9% (4.7) FEF_25‐75_, 35.6% (8.8) R5‐R20, 39.3% (5.9) AX. Thirty‐one patients were receiving daily inhaled corticosteroid (ICS)/long‐acting β2‐agonist (LABA) therapy, and seven were receiving triple therapy. The mean beclomethasone‐equivalent ICS dose was 1720 μg (84.5). All patients withheld their medications prior to testing.

Across the overall cohort, no significant correlation was observed between PD_10_ and BDR in FEV_1_, whether BDR expressed as absolute change (*r* = −0.137, NS), percentage predicted change (*r* = 0.040, NS) or as log‐transformed values (absolute change *r* = 0.052, % predicted change *r* = 0.057, all NS). These findings indicate no linear association between AHR and BDR (Figure 1). When assessing BDR in relation to small airway function, FEF_25‐75_, AX and R5‐R20 showed no significant correlations (*r* = −0.064, *r* = −0.190, *r* = −0.154, all NS).

**FIGURE 1:**
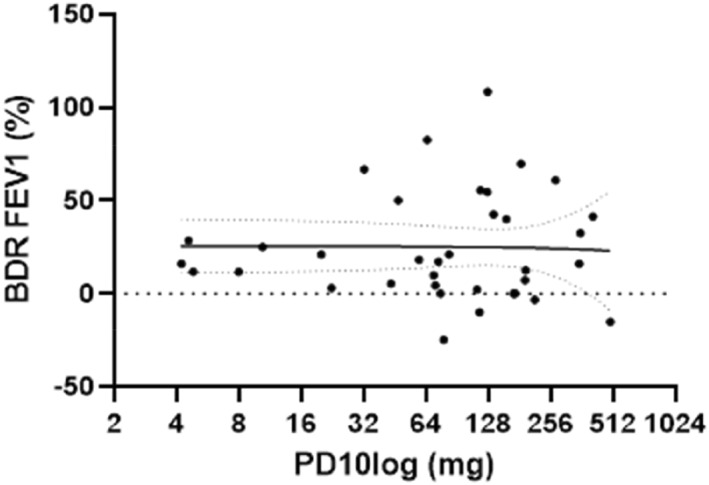
Scatter plots of the mannitol PD10 against the BDR (*r* = −0.039, NS) showing linear regression line and 95% CI. BDR: bronchodilator response, FEV1: forced expiratory volume in 1 s, PD10: provocative mannitol dose causing a 10% decrease in FEV1.

This lack of association with PD10 persisted when different BDR thresholds were applied (> 5%: *r* = −0.007; > 10%: *r* = −0.014, ≥ 12% *r* = −0.219, all NS). Analysis according to T2 biomarkers found no significant correlations between PD10 and BDR in regard to FeNO (≥ 50 ppb: *r* = −0.114, < 50ppb: *r* = −0.317, both NS) or blood eosinophils (≥ 500 cells/μL: *r* = −0.041, < 500 cells/μL: *r* = −0.239, both NS).

There was also no correlation between PD10 and FEV_1_, FEF_25‐75_, R5‐R20 and AX at baseline (*r* = 0.280, *r* = 0.329, −0.287, −0.280, all NS). Furthermore, the non‐significant correlations persisted when analysed in the subcohort within the normal range (FEV_1_ ≥ 80%, FEF_25‐75_ ≥ 60%, R5‐R20 < 0.1 kPa/L/s, AX < 1.0 kPa/L), and the pathological range.

Our results reinforce the notion that BDR to salbutamol and AHR to mannitol reflect distinct facets of airway pathophysiology in severe, uncontrolled asthma. BDR reflects ASM relaxation influenced by baseline geometry, whereas AHR to indirect stimuli evaluates broader mechanisms, including T2 inflammation and structural changes [3]. It also explains perhaps why some patients demonstrate marked AHR despite minimal BDR, and vice versa [7].

Only a few studies have examined the correlation between BDR and AHR, and most have reported no or only weak correlations. Most studies assessed AHR using direct bronchial challenge tests with methacholine or histamine and were restricted to patients with mild‐to‐moderate asthma or individuals exhibiting AHR without a formal diagnosis of asthma [1, 7, 8]. The only study assessing BDR and AHR using an indirect challenge used adenosine 5′ monophosphate (AMP) and was performed in children with mild‐to‐moderate asthma [8]. AMP moderately correlated with BDR, while methacholine showed a weak correlation.

Consequently, disease activity in severe asthma should be evaluated not on a single physiological test, but on a detailed evaluation incorporating BDR and AHR, alongside T2 biomarkers, symptom and exacerbation burden and measures of small airway function. This approach permits to better capture the multidimensional nature of asthma [1, 7]. This disconnect between biomarkers and AHR has also been shown to be consistent in biologic treatment in severe asthma [9].

We acknowledge some limitations. Patients were receiving inhaled therapies, which may have affected both BDR and AHR responses despite standardised testing conditions. We performed only indirect challenge testing, in that direct challenge would be more closely aligned with ASM. Moreover, these retrospective analyses were obtained from studies conducted at a single centre in a relatively small cohort, and a more heterogeneous sample would have been achieved from a wider patient population.

In summary, BDR and AHR represent complementary yet distinct processes. Their lack of correlation in severe asthma reinforces the need for a nuanced, multidimensional approach to functional assessment in both clinical practice and research.

## Author Contributions

**Philipp Suter:** investigation, methodology, formal analysis, writing – original draft, visualization. **Robert Greig:** formal analysis, writing – review and editing. **Rory Chan:** writing – review and editing. **Brian J. Lipworth:** conceptualization, writing – review and editing.

## Funding

The authors have nothing to report.

## Ethics Statement

Ethical approval for this work was not required, as it involves secondary analysis/reporting of data from studies (EudraCT 2019‐003763‐22 and EudraCT 2021‐005593‐25) that had already obtained ethical approval from the appropriate institutional review boards/ethics committees. All original studies were conducted in accordance with the Declaration of Helsinki and applicable regulatory requirements.

## Conflicts of Interest

Philipp Suter reports a relationship with AstraZeneca UK Limited that includes: speaking and lecture fees. Philipp Suter reports a relationship with GSK that includes: speaking and lecture fees. Philipp Suter reports a relationship with Lung League Fribourg (Switzerland) that includes: funding grants without influence on work reported in this paper. Philipp Suter reports a relationship with Swiss Lung Foundation (Switzerland) that includes: funding grants without influence on work reported in this paper. Robert Greig reports a relationship with AstraZeneca UK Limited that includes: speaking and lecture fees and support attending meetings (BTS). Rory Chan reports institutional grants awarded from Asthma + Lung UK, Chiesi, AstraZeneca and GSK; serving on advisory boards for AstraZeneca and Vitalograph; personal fees (talks and/or draughting educational material) from AstraZeneca, Chiesi, Thorasys and Vitalograph; and support attending meetings from AstraZeneca, Chiesi, NIOX, Sanofi‐Regeneron and Vitalograph. Brian J Lipworth reports a relationship with AstraZeneca that includes: consulting advisory boards, grants, and lectures; Sanofi‐Genzyme/Regeneron that includes attending meetings, advisory boards and lectures; Chiesi that includes: consulting, grants, lectures, and attending meetings; Lupin that includes: consulting; Glenmark Pharmaceuticals Limited that includes: consulting and lectures; Sandoz UK Ltd that includes: consulting; Vitalograph and Thorasys that includes equipment The son of Dr Brian Lipworth is presently an employee of AstraZeneca.

## Data Availability

The data that support the findings of this study are available from the corresponding author upon reasonable request.
